# North/South Differences Among Italian Emerging Adults Regarding Criteria Deemed Important for Adulthood and Life Satisfaction

**DOI:** 10.5964/ejop.v12i2.1078

**Published:** 2016-05-31

**Authors:** Giovanni Piumatti, Maria Garro, Laura Pipitone, Angela Maria Di Vita, Emanuela Rabaglietti

**Affiliations:** a Institute of Social Medicine, Faculty of Medicine, University of Belgrade, Belgrade, Serbia; b Department of Psychology, University of Turin, Turin, Italy; c Department of Psychology, University of Palermo, Palermo, Italy; University of Liverpool, Liverpool, United Kingdom

**Keywords:** criteria for adulthood, education satisfaction, emerging adults, Italy, life satisfaction, transition to adulthood

## Abstract

The main goal of this study was to compare Northern and Southern Italian emerging adult university students, regarding the importance attributed to criteria for adulthood and the levels of life and education satisfaction. Self-report questionnaires were filled by 475 Northern and Southern Italian University students (Age M = 22.91, 76% females, n = 359). Multivariate analysis of variance revealed that Southern emerging adults were more likely to place importance on family capacities, norm compliance, interdependence and role transitions as criteria for achieving adulthood than Northern emerging adults. Regarding gender differences, females were more likely to believe in the importance of norm compliance than males, while males were more likely to espouse the importance of legal transitions. Finally, emerging adults from the North reported higher levels of life satisfaction than their Southern counterparts. We interpreted these findings in light of socio-economical and gender socialization differences among Northern and Southern Italian emerging adults.

In the past, transition to adulthood in Western cultures was marked by life events such as graduation, beginning a career and marriage (Modell, Furstenberg, & Hershberg, 1976). Nowadays, similarly to the rest of Europe (Eurostat, 2009), or the US and Canada (Arnett, Ramos, & Jensen, 2001; Cheah, Trinder, & Gokavi, 2010), the mean age of first marriage for females in Italy increased from approximately 24 in 1975 to 31 in 2011, and from 27 to 34 for males (ISTAT, 2012). At the same time, each year, Italian university students account for a good proportion of high school graduate students (ISTAT, 2009), while youth unemployment (18-24) has recently reached a new all-time high of 41.2% (ISTAT, 2014). In this current scenario, Italian young adults have more time to experiment with different life possibilities and as a result experience an extended period of transition known as *emerging adulthood* (Arnett & Tanner, 2006).

The construct of emerging adulthood is embedded in socio-cultural contexts that delay adult responsibilities, often through extended post-secondary education (Arnett & Galambos, 2003). This transition phase is shorter in cultures that encourage people to marry or make other life decisions, such as career choice, at a younger age (e.g., Nelson, 2003; Nelson, Badger, & Wu, 2004). On the contrary, in the absence of social safety valves, the transitions become “a combined developmental undertaking” of parents and sons/daughters (Scabini & Cigoli, 2000, p. 142; Cuconato, 2011). In particular, environments that have fewer career and educational options for emerging adults tend to give greater emphasis on family obligations, especially in strong collectivistic cultural contexts (Fuligni & Pedersen, 2002). In this sense, the North and the South of Italy represent the opposite sides of a coin with regard to youth employability rates, higher education attainment, generational transitions and family formation process (ISTAT, 2012). In the North, the higher youth employability and education attainment (especially among females) and a later transition to marriage with respect to the South, favor modern family behaviors (living alone before marriage, a large number of consensual unions) (Avola, 2009; Santarelli, 2011) and loose psychological and economical bonds with the family of origin at younger ages (Piumatti, Pipitone, Di Vita, Latina, & Rabaglietti, 2014). Instead, in the South of Italy, family represents not only the sphere of affections but also a safety valve, the “undisputed and beloved cradle of new generations” (Bava, 2010, p. 92), a stronger institution than those in which other Italian young people live. Thus, emerging adults from the North of Italy might experience this transition differently than emerging adults from the South on the basis of context-related circumstances regarding specific modes of family functioning and different socio-economical opportunities (Scabini, Regalia, & Giuliani, 2007).

These existing differences between the North and the South of Italy can be outlined in the contraposition between individualistic and collectivistic societies’ values. The terms individualism and collectivism have been used to refer to value systems existing within and across cultural or national groups (Rothbaum & Trommsdorff, 2007). More specifically, according to Triandis (1995), individualism is a social pattern that consists of loosely linked individuals who view themselves as independent of collectives and who give priority to their personal goals over the goals of others. On the other hand, collectivism is a social pattern consisting of closely linked individuals who see themselves as part of one or more collectives (family, coworkers, nation) and are willing to give priority to the goals of these collectives over their own personal goals (Triandis, 1995). This conceptual and cultural framework has also been adopted to describe different traditions of transmission of norms and values from parents to children (e.g., Tamis-LeMonda et al., 2008). Thus, reading from the research literature on individualism vs. collectivism, we can argue how in the North of Italy family efforts may be especially centered on the realization of the individual in the society, reflecting parents’ beliefs about personal choice, intrinsic motivation, self-esteem and self-maximization as the fundamental requirements for children’s successful achievement of autonomy (Walsh, 2012). Conversely, in contexts such as the South of Italy, parents may give greater emphasis to the promotion of relatedness and interdependence in their children, promoting in them values related to connection to close relationships, orientation to the larger community, respect and obedience of cultural and societal norms (Triandis, 1995).

Despite all these contrapositions between Northern and Southern Italy, there is little research on potential differences in the development of emerging adults from these two regional areas. In addition, although numerous studies in North (e.g., Nelson & Barry, 2005; Cheah et al., 2010), and South (e.g., Facio, Resett, Micocci, & Mistrorigo, 2007) America as well as in North (Kins & Beyers, 2010) and West (Nelson, 2009) Europe have examined the criteria emerging adults (aged 18 to 28) consider important to achieve adulthood, less work has been done to examine what criteria are considered important to achieve adulthood among emerging adults in Southern European countries such as Italy (Crocetti et al., 2015; Petrogiannis, 2011). Accordingly, this study examined potential North/South differences among emerging Italian adults university students in significant developmental tasks as reflected in their beliefs about the criteria for adulthood, life, and educational track satisfaction.

## Criteria for Adulthood

According to Arnett (2001) – from a subjective point of view – adulthood is much more strongly defined by young people’s growing tendency to take responsibility for one’s actions, the capacity to accept family responsibilities and compliance with social norms. The endorsement of these and other criteria (e.g., going through legal and biological transitions involving age boundaries and physical growth respectively) among emerging adults would ideally describe a person who is facing the responsibilities of adult life. In particular, researchers (see Arnett, 2003) have documented that contemporary emerging adults, consistently across cultures, tend to view the following four major criteria as necessary and important for adulthood: (1) being independent and self-reliant; (2) being able to form mature relationships; (3) being able to comply with societal norms; and (4) being able to provide and care for a family. Thus, following the theory of emerging adulthood, positively adjusted emerging adults are in the process of becoming less self-oriented and ready to commit themselves to enduring relationships with others by showing a predisposition to be involved in relations and maintaining a strong focus on their actions and goals (e.g., Arnett, 2003; Isaacs, Soglian, & Hoffman, 2015; Nelson & Padilla-Walker, 2013). The transition towards the endorsement of other-related criteria for adulthood would take place with ageing, earlier for females than for males, and with the increasing experiencing of independent living conditions, a certain degree of economic self-subsistence and long term relationships (e.g., Cheah, Trinder, & Gokavi, 2010; Kins & Beyers, 2010; Lanz & Tagliabue, 2007; Nelson & Barry, 2005; Piumatti, Giannotta, Roggero, & Rabaglietti, 2013). Concurrently, mainly transition-linked increases (e.g., leaving the parental home, finishing education, getting a job) may contribute to the endorsement of certain criteria (e.g., family responsibilities when getting married, interdependence when starting a new relationship, independence when getting a job) and accompany increases in psychological well-being such as positive life-satisfaction (Galambos, Barker, & Krahn, 2006; Galambos & Krahn, 2008; Kins & Beyers, 2010).

Similarly to emerging adults in the United States, Argentina, Austria, Greece and Australia (Facio et al., 2007; Nelson & Barry, 2005; Petrogiannis, 2011; Sirsch, Dreher, Mayr, & Willinger, 2009; Weier & Lee, 2015), Italian emerging adults have showed high levels of consent regarding the defining features for adulthood as described above (Piumatti & Rabaglietti, 2015). They seem to embrace the individualism of the Western, North-European and American culture, and at the same time to reflect the more traditional cultural values of family obligations and consideration for others. Such results evidence that different cultures share a common view regarding the markers of adulthood. Nevertheless, these criteria also consistently appeared to vary according to group values, ideologies and socio-cultural environments, even within Western societies (e.g., Arnett, 2003; Arnett & Galambos, 2003). In sum, while the attention toward these criteria recur among emerging adults in many nations and cultures, socio-political and economic characteristics of a country as well as cultural beliefs and norms about social relationships may affect the importance addressed to specific criteria in any given context. However, one socio-cultural comparison that has received little attention is between emerging adults from different socio-economical contexts within the same country, such as the North and the South of Italy. As subsequently discussed, different regional experiences regarding economical and available working opportunities within the same cultural context may lead to different attitudes with regard to emerging adulthood concerns.

## North/South Differences

It has been previously noticed how emerging adults from the North of Italy gave particularly high importance to aspects of personal and professional growth (e.g., Scopesi & Bertani, 2003). In fact, they seemed more oriented to fulfill themselves in the profession for which they studied and to focus their attention more on individualistic goals such as self-realization at work rather than giving more weight to personal relationships. Accordingly, by the means of satisfaction regarding life and study choice, young adults from the North have reported in previous studies (Amerio, 2010) to appreciate their post-secondary study choices and to positively evaluate the fulfillment of future career plans. Moreover, it would seem that Northern Italian young adults continue living with their parents until they have reached a stable position in the job market and a satisfactory economic independence (Alfieri, Sironi, Marta, Rosina, & Marzana, 2015; Briulotta, 2009). Nonetheless, they also tended to value issues related to family life, love and friendship, confirming a “growing tendency toward the areas of restricted social relationships” (de Lillo, 2007, p. 140). Indeed, it is important to point out that the support received from the family of origin is a significant aspect also in the North of Italy (Piumatti et al., 2014). In addition, as noticed by Paleari, Rosnati, and Lanz (2002) in a study conducted on entire family nuclei from the North (i.e., including immediate and extended family members), the support received from one’s family played an essential role in the psychological well-being of young adults.

When facing a stagnant job market, young people especially in search of their first occupation are obliged to rely upon the support of their family or on their partner, with the risk of perpetuating social and economic inequalities. At this regard, Palidda (2002) stated that most Italian young Southern residents consider young adulthood as an uncertain season in which to have low-qualified, badly paid and irregular job experiences. Without concrete forms of support from their families young people are thus forced to curtail their aspirations or to postpone them instead of nurturing the hope to fulfill their future plans. In order to express a positive attitude toward their future independent lives, emerging adults from the South of Italy have to leave behind part of their perception of being dependent on the support they receive in their parents’ household. As they find difficulties in moving forward along their pathway toward adulthood, they may keep investing more energy in strengthening those parts of their lives where they receive the greatest amount of support for their activities. In the case of Southern Italian emerging adults, this seems to be their families (Inguglia, Ingoglia, Liga, Coco, & Cricchio, 2015). Moreover, reading from Musumeci (2009), for graduates from the South it is more complicated to plan and express preferences for a particular profession (and therefore appreciation for a university course that may lead to a specific type of profession) because the reality of a stagnant job market forces them to adapt to and accept whatever job they may find. In this socio-economic climate, the family of origin represents one of the few certainties on which to base one’s everyday life and value system. More specifically, the role of the family is determinant for finishing education and subsequently finding a suitable job. In addition, for young adults from the South, investing in family relationships represents a resource for facing the dissatisfaction that the sphere of work may procure. In this way, the life of a couple and the investment in the relationship may allow young people from the South to compensate for occupational and economic instability (Musumeci, 2009).

Overall, the striking differences in experiencing the transition to adulthood whether one is living in the North or in the South of Italy have been previously documented by looking at individual psychological outcomes such as well-being, feelings of fulfillment in life or perceived support (see Piumatti et al., 2014). Drawing from such conclusions and in light of the still existing inequalities within the Italian context, the current study aimed at further investigating how the perceptions of the criteria for adulthood may differ between Northern and Southern Italian young adults.

## Aims and Hypotheses

The main goal of this study was to compare Northern and Southern Italian emerging adult university students, regarding the importance attributed to criteria for adulthood and the levels of life and education satisfaction. Despite the merely descriptive nature of the study, and the fact that no previous research has examined the differences between emerging adults from the North and the South of Italy specifically regarding their endorsement of the criteria for adulthood, building upon the features of this time period as proposed in Arnett’s theory of emerging adulthood we formulated the following hypotheses: (1) first, we predicted that Italian emerging adults from the South would place more importance on interdependence, family capacities and more traditional and externally identified criteria (e.g., role transition) as important markers of adulthood, than their peers from the North; (2) second, as suggested by previous research (Ciairano, Rabaglietti, Roggero, & Callari, 2010; Piumatti et al., 2014), we expected the latter to report higher levels of satisfaction in life also with regard to their post-secondary educational track than the former. Moreover, a complementary goal of this study was to investigate gender-related differences in endorsement of adulthood criteria among Italian emerging adults. Accordingly, reading from past research (Gilligan, 1982; Josselson, 1996; Mayseless & Scharf, 2003; Montgomery, 2005), socialization that emphasizes certain criteria for either men or women may lead to gender differences in criteria for adulthood, we further hypothesized that female participants would be more oriented towards the consolidation of the relationships other-oriented than male participants and to place greater importance on criteria regarding interdependence and family capacities as well as norm compliance.

## Method

### Participants

The sample used in the current study consisted of 475 Italian native graduate and undergraduate University students (76% females, *n* = 359) recruited from the University of Turin in Piedmont a North-western region of Italy and the University of Palermo in the Southern region of Sicily. Both universities are public institutions. The criteria to include participants in this research was age, in a range of 18 to 30 years old, the condition of University student at the time of data collection, and being born in the same macro-region where the University was located (i.e., North or South of Italy). The mean age of the entire sample was 22.91 years (*SD* = 2.71; age ranged from 18 to 30). Northern participants came from families where 44% of mothers and 55% of fathers had obtained at least a diploma of secondary education (high school or professional school diploma), compared to 44% of mothers and 47% of fathers of Southern participants. All participants were unmarried and childless. Most participants came from middle class families, with 56% of Northern participants and 59% of Southern participants considering the general economical conditions of their families of origin as belonging to the national average.

### Procedure

Participants were recruited through an announcement of the study in undergraduate and postgraduate courses. They completed a questionnaire via the Internet. Informed consent was obtained online, and only after consent was given could the participants begin the questionnaires.

The computer-administered questionnaire took approximately 30 minutes to complete at which point the purpose of the study and expected results were provided to the participants. The Italian versions of the scales included in the questionnaire were created by translating and back translating them by English native speakers.

### Measures

#### Importance of Criteria for Adulthood

Participants rated the importance of 36 criteria for adulthood (e.g., Arnett, 1998, 2003) on their degree of importance on a scale of 1 (not at all important) through 4 (very important). Based on previous research (e.g., Arnett, 2001, 2003), the criteria were then grouped into six categories including *interdependence* (5 items; e.g., “Committed to long-term love relationship”), *role transitions* (6 items; e.g., “Have at least one child”), *norm compliance* (8 items; e.g., “Avoid becoming drunk”), *age/biological transitions* (4 items; e.g., “Grow to full height”), *legal transitions* (6 items; e.g., “Have obtained license and can drive an automobile”) and *family capacities* (8 items; e.g., “Become capable of caring for children”). The organization of the subscales was obtained through a theory based approach rather than by a quantitative statistical approach such as factor analysis (Arnett, 2001). Specifically, the items of the family capacities subscale were all drawn from the anthropological literature, which has identified gender-specific criteria used in many traditional cultures as criteria for the transition to adulthood (Gilmore, 1996). Similarly, the items on the role transitions subscale were drawn from sociological literature, which has long used a series of specific role transitions as the defining criteria for the transition to adulthood (Goldscheider & Goldscheider, 1999; Hogan & Astone, 1986). The analysis of internal consistency pointed out that subscales’ alpha levels in our sample were considerably better than in previous studies (e.g., Arnett, 2003; Nelson & Barry, 2005; Sirsch et al., 2009): interdependence, Cronbach’s *α* = .60; role transitions, *α* = .82; norm compliance, *α* = .84; age/biological transitions, *α* = .71; legal transitions, *α* = .82; family capacities, *α* = .89. Indeed, results of reliability analyses on these subscales have always been moderate or low in past researches (e.g., Arnett, 2003; Nelson & Barry, 2005). Nevertheless, these measures have been used in a variety of previous studies (e.g., Arnett, 2003; Nelson, 2009) indicating how researchers agree upon the fact that although statistical evidence for Arnett’s conceptual model of criteria for adulthood might lack, this model shows high face validity (e.g., Barker & Galambos, 2005). Thus, we considered our reliability results as acceptable.

#### Life Satisfaction

Life satisfaction (*α* = .70) was assessed with an adaptation of the five-item Satisfaction with Life Scale (Diener, Emmons, Larsen, & Griffin, 1985). The items (e.g., “I am satisfied with my life”, “In most ways my life is close to my ideal”) were rated on a 4-point Likert-type scale ranging from 1 (I totally disagree) to 4 (I totally agree).

#### Education Satisfaction

Adolescents’ school satisfaction after the transition to post-comprehensive schooling (*α* = .73) was measured by the four-item Satisfaction with Educational Track scale (Nurmi, Niemivirta, & Salmela-Aro, 2003). The items (e.g., “How satisfied are you with your educational track?”, “Do you enjoy going to school?”) were rated on a 5-point scale ranging from 1 (not at all) to 5 (very much).

## Results

### Data Analysis

Before proceeding to analyze the data, all the scores for the items for each subscale were examined for the accuracy of data entry, detecting and replacing missing values, identifying univariate and multivariate outliers. We also examined the data for detecting multicollinearity and non-normal distribution among dependent variables. The main analyses consisted in multivariate analysis of variance (MANOVA) and post-hoc univariate analyses of variance (ANOVA) were carried out to look at differences in importance addressed to the criteria for adulthood, life satisfaction and education satisfaction depending on participants’ provenience (North vs. South) and gender. In accordance with Cramer and Bock (1966), MANOVA was first performed on the means for each criterion’s category to help protect against inflating the Type 1 error rate in the follow-up ANOVAs and post-hoc comparisons. However, prior to conducting the MANOVAs, a series of Pearson correlations were performed between all of the dependent variables in order to test the MANOVA assumption that the dependent variables would be correlated with each other in the moderate range (i.e., .20 - .60; Meyers, Gamst, & Guarino, 2006).

### Preliminary Analysis

No transformation of the distributions was found that can accommodate the model well. We considered acceptable values of skewness and curtosis between -1 and 1 (see Muthén & Kaplan, 1992). According to these general principles, the assumption of the normality of the distribution for the variables of our study was met, with values of skewness and curtosis ranging respectively from -.68 to .45 and from -.71 to .70. Given the significant low rates of missing values on each item of the scales (less than 3%), the expectation-maximization algorithm was used for data imputation, a robust method to obtain maximum likelihood estimates (Schafer, 1997). This decision was also made as no systematic correlation was detected between these missing values and the scores of other variables among these subjects (*r* < |.20|) (see Raaijmakers, 1999). Prior to the analysis, data were carefully examined for univariate outliers (classified as scores more than three standard deviations above or below the mean; see Hoaglin & Iglewicz, 1987). Accordingly, no case was excluded from further analysis.

As can be seen in Table 1, a meaningful pattern of correlations was observed amongst most of the dependent variables, suggesting the appropriateness of MANOVAs. These preliminary results should not indicate any problems in terms of dependent variables’ multicollinearity (Field, 2009). Additionally, the Box’s M values of 92.82 and 14.41 were associated with a *p* value respectively of .02 and .11, which was interpreted as non-significant based on Huberty and Petoskey’s (2000) guideline (i.e., *p* < .005). Therefore, the covariance matrices between the groups were assumed to be equal for the purposes of the MANOVAs. Correlations among subscales regarding criteria for adulthood were small to moderate in magnitude (ranging from *r* = .20, *p* < .01 to *r* = .60, *p* < .01) confirming that these domains in which adult status is expected to be demonstrated, reflect different facets or markers of adulthood. Similar results were obtained in previous studies (e.g., Piumatti et al., 2013; Petrogiannis, 2011). The correlation between life satisfaction and education satisfaction was equal to .22, *p* < .01, confirming that these constructs were describing different facets of satisfaction.

**Table 1:** Bivariate Correlations Among Psychological Study Variables

Measure	1	2	3	4	5	6	7	*M*	*SD*
Criteria for adulthood									
1. Family capacities	-							3.27	.53
2. Norm compliance	.42*	-						3.13	.59
3. Interdependence	.47*	.41*	-					3.01	.46
4. Age/biological transitions	.43*	.28*	.41*	-				2.43	.70
5. Role transitions	.49*	.41*	.44*	.45*	-			2.80	.68
6. Legal transitions	.26**	.20*	.37*	.60*	.37*	-		1.96	.69
Satisfaction									
7. Life satisfaction	.03	.05	.02	.12*	.06	.06	-	2.99	.60
8. Education satisfaction	.01	.13*	.19*	-.04	.03	-.02	.22*	3.66	.67

*p < .01.

### Results of MANOVA and ANOVAs

Two MANOVAs were conducted to determine whether importance addressed to the criteria for adulthood and life satisfaction and education satisfaction differed as a function of sub-group membership (North vs. South) and gender. Prior to this, t-test for independent samples and *χ*^2^-tests reported that the two sub-groups did not differ according to age and gender compositions. The first MANOVA consisted of emerging adults’ ratings of the importance of criteria for adulthood (family capacities, norm compliance, interdependence, age/biological transitions, role transitions, legal transitions). To evaluate magnitudes of effect sizes we adopted Cohen’s (1988) general guidelines (.01 = small, .06 = medium, .14 = large). Results revealed a significant main effect of gender, Pillais’ Trace = .08, *F*(6, 465) = 6.77, *p* < .001, partial η^2^ = .08, and sub-group membership, Pillais’ Trace = .08, *F*(6, 465) = 7.13, *p* < .001, partial η^2^ = .08. According to these results of multivariate effect sizes, both gender and sub-group membership respectively explained 8% of the variance in the canonically derived dependent variable. Prior to conducting a series of follow-up ANOVAs, the homogeneity of variance assumption was tested for all the subscales. Based on a series of Levene’s *F* tests, the homogeneity of variance assumption was considered satisfactory. A series of one-way ANOVA’s on each of the six dependent variables was conducted as a follow-up test to the MANOVA. Results determined that female participants (*M* = 3.17, *SD* = .57) placed greater value on norm compliance as a criterion for adulthood than males (*M* = 2.99, *SD* = .61). On the contrary, male participants (*M* = 2.19, *SD* = .74) were more likely to rate legal transition important for achieving adulthood than females (*M* = 1.88, *SD* = .65). Southern participants emphasized the following criteria for adulthood more than the Northern participants: family capacities, (South: *M* = 3.35, *SD* = .52; North: *M* = 3.22, *SD* = .53), norm compliance, (*M* = 3.31, *SD* = .53; *M* = 3.01, *SD* = .59), interdependence, (*M* = 3.07, *SD* =.45; *M* = 2.98, *SD* = .46), and role transitions, (*M* = 2.97, *SD* = .62; *M* = 2.97, *SD* = .62) (see Table 2).

**Table 2:** One-Way ANOVAs With Criteria for Adulthood, Life Satisfaction and Educational Track Satisfaction as Dependent Variables and Sub-Group Membership as Independent Variable

Dependent Variable	Levene’s		ANOVAs			North		South	
	*F*	*p*	*F*	*p*	η^2^	*M*	*SD*	*M*	*SD*
Criteria for adulthood									
Family capacities	.09	.771	7.40	.007	.005	3.20	.52	3.35	.52
Norm compliance	2.16	.142	29.69	.000	.045	3.01	.59	3.31	.53
Interdependence	.55	.460	4.91	.027	.007	2.98	.46	3.07	.45
Age/biological transitions	.08	.777	.01	.959	.001	2.43	.70	2.43	.70
Role transitions	5.84	.160	19.91	.000	.034	2.70	.69	2.97	.62
Legal transitions	1.57	.195	.01	.959	.003	1.96	.69	1.97	.69
Satisfaction									
Life satisfaction	.33	.566	7.06	.008	.011	3.05	.58	2.90	.61
Education satisfaction	1.03	.381	1.22	.268	.003	3.63	.71	3.70	.61

*Note. N* = 475; η^2^ = Partial eta squared.

The second MANOVA consisted of emerging adults’ reports on life and educational track satisfaction. Results revealed a significant main effect of sub-group membership, Pillais’ Trace = .02, *F*(2, 469) = 4.35, *p* < .05; partial η^2^ = .02, implying that 2% of the variance in the canonically derived dependent variable was accounted for by sub-group membership. Also in this case, based on a series of Levene’s *F* tests, the homogeneity of variance assumption was considered satisfied. No interaction effects were found to be significant. Based on ANOVAs post hoc analyses, it was determined that Northern participants (*M* = 3.05, *SD* = .58) reported higher levels of life satisfaction than Southern participants (*M* = 2.90, *SD* = .61) (see Table 2).

## Discussion

We hypothesized that (1) emerging adults from the South of Italy would place more importance on interdependence, family capacities and more traditional and externally identified criteria (e.g., role transition) as important markers of adulthood, than their peers from the North; that (2) emerging adults from the North would report higher levels of satisfaction in life also with regard to their post-secondary educational track than their peers from the South; and that (3) females would be more oriented towards the consolidation of other-oriented relationships than males and would place greater importance on criteria regarding interdependence and family capacities as well as norm compliance. In sum, all our hypotheses were confirmed by our results, given the exception of the North/South difference regarding educational track satisfaction that was similar across groups. At this stage of their lives, Northern and Southern Italian emerging adults attending University may indeed equally place hope on the fact that trough achievements in higher education they can fulfill their future aspirations. Accordingly, they exhibit high levels of satisfaction for the education track they choose to pursue.

### North/South Differences

As expected, traditional and external markers such as transitional events and roles including being married or settling into a long-term career were considered as more important indicators of adulthood among emerging adults from the South, which may reflect a more conventional socialization regarding what it means to be an adult (Arnett, 2001). The ages of marriage and childbearing among emerging adults in the South are lower than that found in the North, and are more likely viewed as important role transitions. Similarly, Southern emerging adults were also more likely to endorse family capacities, norm compliance and interdependence markers as important for having achieved adulthood. Overall, these results confirm our hypotheses of deep differentiations between Northern and Southern Italian emerging adults in the way that they perceive and depict adulthood. The former appear to embody a more individualistic approach to this transition phase and perhaps are more focused on self-related goals such as the acquisition of individual practical skills. Comparatively, the latter evidence a marked predisposition towards multiple collectivistic facets of adulthood, regarding both personal relationships (e.g., committing to long term relationships, getting married, maintaining good relationships with parents) and respecting societal norms and values (avoid the use of alcohol and drugs). However, in line with our hypotheses, emerging adults from the North reported being more satisfied with their lives in relation with their Southern peers. Thus, despite the fact that in the South emerging adults seem to embrace the values and criteria for adulthood sooner than in the North, this condition does not imply they are also exhibiting higher levels of well-being. At this stage, other individual differences may count, for example reaching economic independence or better professional future perspectives. Overall, we can read that among young Southern adults there is a stronger appreciation of family and relationship values in light of their necessity to rely more on the informal, but functional, supporting network of family and relatives in the areas of work and education (Palidda, 2009). In addition, given that most young adults have high expectations to find a steady job and reach economic stability (Salmela-Aro & Tynkkynen, 2010) in contexts where it turns out to be more difficult to settle or to emancipate themselves from the family of origin, young adults may negatively suffer the impossibility to contribute to the economy of the household they share with their parents (Manacorda & Moretti, 2006). A condition that could in turn represent a source of perceived stress for all family members as it precludes chances of future emancipation and realization of personal goals (Togliatti, 1996). Instead, young adults from the North of Italy may envision their future in a more individualistic way, accepting to be either fully or partially beholden to their parents for receiving the support they need while they complete their education and build their position in the labour market (Berlin, Furstenberg, & Waters, 2010). In sum, such results are aligned with previous research that highlighted how among emerging adults different perceptions of what are the important criteria for reaching adulthood can be explained by individualistic or collectivistic societies’ values (Badger, Nelson, & Barry, 2006).

### Gender Differences

The socialization explanation for the endorsement of criteria for adulthood seems to hold also with regard to gender differences found in this study. In fact, as expected, females reported a stronger inclination towards other-related criteria than males, namely complying with societal rules and norms. On the other hand, males gave more importance to the freedom and allowance of certain habits such as smoking and drinking. As noted previously, because of the competition to complete education and find a job, young men especially may be at risk of a period of emerging adulthood and adulthood filled with problematic behaviors (Nelson, Duan, Padilla-Walker, & Luster, 2013; Wilsnack, Vogeltanz, Wilsnack, & Harris, 2000). Instead, other-related criteria such as norm compliance are more relevant for young women in particular during the age period of emerging adulthood, in which they start to consider the possibility of forming a family sooner than their male counterparts (Cinamon & Rich, 2014).

## Limitations and Implications for Future Research

This study was not without limitations. As noted previously, the descriptive nature of the analyses precludes causal inferences. Specifically, longitudinal studies will evidence how in correspondence with crucial life events (e.g., leaving the parental home, getting married, starting a new job) emerging adults from the North and the South of Italy will direct their individual transitions’ trajectories regarding adulthood’s self-perception and well-being. Another limitation is that participants included only University students. More research is needed to increase knowledge about individual differences in young people who do not attend University after high school, especially on issues related to criteria for adulthood, attitudes and beliefs about marriage and family life and identity formation. Finally, future work will need to include other important characteristics for this age period of transition – whether behavioral, relational, or intrapersonal – that might contribute to define context related features of the transition to adulthood.

Despite these weaknesses, findings from this study may have implications for future research. In particular, the specific effects of discrepancies between parents and emerging adults on the criteria deemed necessary for adulthood should be examined both in the North and the South of Italy. In fact, research suggests that family conflict increases when young people do not conform with parental expectations for adulthood (Roest, Dubas, Gerris, & Engels, 2009; Schnaiberg & Goldenberg, 1989) and decreases as young people achieve *child-endorsed* criteria for adulthood (Shulman & Ben-Artzi, 2003). Furthermore, research has shown that the historically rooted industrial versus agriculture orientation of the North and South of Italy, relates to the psychology of caregivers and to their socialization attitudes and behaviors (Barni, Alfieri, Marta, & Rosnati, 2013; Bornstein, 1994; Bornstein, Venuti, & Hahn, 2002; Hoff, Laursen, & Tardif, 2002) and that Italian’s living in these two different regions tend to differ in their general feelings towards family and society. Therefore, in the contexts where the new timetable for adulthood has created a stronger sense of entitlement and a lingering pattern of dependency to the family of origin, differences in parent–child values regarding criteria for adulthood may result in an increase in parent–child conflict during the emerging adult years (Berlin, Furstenberg, & Waters, 2010; Zarit & Eggebeen, 2002).

## Conclusions

Despite the limitations, this study makes several unique contributions. Most notably, it is the first study that examines criteria for adulthood comparing participants from the North and the South of Italy. Thus, the findings provide a significant contribution to our understanding of how emerging adulthood may be structured differently in different socio-economical contexts present within the same national and cultural setting. In particular, it contributes to stress out how actual is from a developmental perspective the topic of identity formation during the transition to adulthood, giving credit once again to the theory of emerging adulthood as it has been elaborated by Arnett (2001). The contraposition between Northern and Southern Italian emerging adults is in fact a discrepancy between different ways of coping with adverse economical and structural conditions that are transversally present in Italy although with local specificities. Such conditions are very much similar to those experienced by young adults in other European countries where the backlashes of the economic crisis are still evident. At this regard, in order for us to understand from a psychological point of view how individuals go through these delicate periods of transitions, such as the one from education to work, and face challenges and opportunities in their environments at a large, the inclusion of context-related variables is fundamental. In this way we will grow our knowledge about positive and less positive young adults’ developmental pathways in terms of self-perceived well-being and identity construction. Moreover, we may further contribute to shed light on the determinants of personal decisions regarding family formation processes, living habits and even migrations during this age period.
